# The clinical impact of the COVID-19 pandemic on daily urological practice: first 3-month multicenter results from İstanbul

**DOI:** 10.3906/sag-2009-184

**Published:** 2021-06-28

**Authors:** Mehmet Çağlar ÇAKICI, Mustafa Zafer TEMİZ, Ayberk İPLİKÇİ, Faruk ÖZGÖR, Alper Kerem AKSOY, Murat ÖZER, Selçuk ERDEM, İsmail ULUS, Eyüp Veli KÜÇÜK, Alper ÖTÜNÇTEMUR, Enes DEĞİRMENCİ, Reşat AYDIN, Gökhan ATIŞ, Ahmet Yaser MÜSLÜMANOĞLU, Ömer SARILAR, Faruk ÖZCAN, Asıf YILDIRIM

**Affiliations:** 1 Department of Urology, Istanbul Medeniyet University Goztepe Training and Research Hospital, İstanbul Turkey; 2 Department of Urology, Health Science University, Bağcılar Training and Research Hospital, İstanbul Turkey; 3 Department of Urology, Health Science University, Sultangazi Haseki Training and Research Hospital, İstanbul Turkey; 4 Department of Urology, Health Science University, Ümraniye Training and Research Hospital, İstanbul Turkey; 5 Department of Urology, Health Science University, Okmeydanı Training and Research Hospital, İstanbul Turkey; 6 Department of Urology, Istanbul Faculty of Medicine, İstanbul University, İstanbul Turkey

**Keywords:** COVID-19 virus infection, outpatients, pandemics, urology, urologic surgical procedure

## Abstract

**Background/aim:**

The aim of this paper was to determine the general tendencies of urology patients and effect of COVID-19 pandemic on daily urological practice at tertiary centers located in the most affected area in Turkey.

**Materials and methods:**

We retrospectively analyzed the data of 39,677 patients (group 1) that applied to 6 different large-volume tertiary centers in İstanbul for outpatient consultation, surgery, or other procedures in the 3-month period between March 16 and June 14, 2020. The distribution of the number of patients who applied to subspecialty sections of urology outpatient clinics and inpatient services were recorded by weeks. That data was compared to data obtained from 145,247 patients that applied to the same centers in the same period of the previous year (group 2). The reflection of worldwide and Turkish COVID-19 case distribution on the daily urological practice was analyzed.

**Results:**

There was a decrease in the number of patients in all subspecialty sections the in group 1 compared to group 2; however, there was a significant proportional increase in urooncology and general urology admissions. A decrease of approximately 75% was observed in the total number of surgeries (p < 0.001). We detected a negative correlation between the numbers of admission to all outpatient clinics and COVID-19 cases or deaths in Turkey (p < 0.05). The same negative correlation was present for all surgical procedures and consultations (p < 0.05). The multivariate linear regression analysis revealed that the number of cases in Turkey, and the number of deaths worldwide affect the number of outpatient clinic admissions (R2 = 0.38, p = 0.028) and urological surgery (R2 = 0.33, p = 0.020) in Turkey negatively.

**Conclusion:**

This novel pandemic has implications even for urology practice. Urological surgical procedures were more affected by COVID-19-related deaths in Turkey and worldwide. Outpatient admissions and urological surgeries decreased significantly by increasing COVID-19 case numbers in Turkey and worldwide deaths.

## 1. Introduction

A novel severe acute respiratory syndrome coronavirus 2 (SARS-CoV-2) emerged in Wuhan City, Hubei Province, China in December 2019 [1]. The virus was determined as the causative agent for a cluster of pneumonia cases initially detected in WuhanWorld Health Organization (2020). Novel coronavirus—China [online]. Website https://www.who.int/csr/don/12-january-2020-novel-coronavirus-china/en/ [accessed 12/01/2020]. . The disease can manifest itself in a wide spectrum, ranging from asymptomatic disease to respiratory failure and death. As of July 1 2020, the number of cases worldwide has exceeded 10 million and the number of deaths has exceeded 500 thousandWorld Health Organization (2020). Coronavirus disease (COVID-19) situation report – 162 [online]. Website https://www.who.int/docs/default-source/coronaviruse/20200630-covid-19-sitrep-162.pdf?sfvrsn=e00a5466_2 [accessed 01/07/2020]. . In Turkey, the number COVID-19 cases has exceeded 200 thousand, while the death toll has surpassed 5150. From the appearance of the first cases in Turkey to the present, 53.74% of the cases were in İstanbulMinistry of Health of Turkey (2020). Current status in Turkey [online]. Website https://covid19bilgi.saglik.gov.tr/tr/ [accessed 28 May 2020]. . The highest number of deaths also occurred in İstanbul.

COVID-19 pandemic has become a global emergency healthcare concern for more than 200 countries, although its center has changed. While the healthcare systems of many countries affected by this crisis can manage the situation, those of some developing countries cannot. Accordingly, a large population with nonpandemic-related health problems cannot receive health care. The global impact of the COVID-19 disease caused by this virus on urological practice has not been clearly established yet. In the affected countries, the downsizing of all urological practice began abruptly in mid-March. Very quickly, all elective procedures were postponed to uncertain date. Some clinicians reported that their outpatient office work was reduced by 40%–80% [2]. General urology practice in our country was quickly affected by this situation as in other specialties. Therefore, the distribution of the number of patients in daily urology practice and the degree of impact on the distribution of patients in urology subspecialties varied. However, whether this effect creates a difference between subspecialties and its relation to the number of COVID-19 cases in our country or worldwide needs to be explained. In this study, we aimed to determine the effect of the COVID-19 pandemic on daily urological practice in İstanbul, where the tertiary centers are located and which coincidentally was the area most affected by the pandemic.

## 2. Materials and methods

The announcement of the study was sent to the busiest urology clinics from tertiary centers across İstanbul via e-mail and social media. This retrospective observational study was conducted after the approval of the Ministry of Health and the Local Ethics Committee (approval date is 24.06.2020 and decision number is 2020/0401) in the departments of urology of 6 different large-volume tertiary centers in İstanbul. The number of outpatient and hospitalized patients, urological surgeries, consultations, and daily interventions were recorded week by week by scanning the hospital registry systems by all urology clinics. All patients who applied to urology clinics in the 3-month period starting from the week following the first case in our country were included in the study. Patients younger than 18 years of age were excluded from the study. In the study, the data of 39,677 patients who applied to our clinics for outpatient service, consultation, surgery, or outpatient procedures such as prostate biopsy or cystoscopy during the 3-month period between March 16 and June 14, 2020 were analyzed (group 1). The distribution of the number of patients who applied to urology outpatient clinics, subspecialty sections of urology outpatient clinics, and inpatient services were recorded by weeks. In addition, consultations, cystoscopy, discharge numbers, the number of patients who had surgery, and the number of frequently performed surgeries were recorded. The reflection of worldwide and Turkish COVID-19 case number distribution on the daily urological practice was analyzed. It was compared to the data from 145,247 patients that applied to the same centers in the same period of the previous year (group 2).

### 2.1. Statistical analysis

The data analysis was performed by using SPSS v. 22 for Windows (SPSS Inc., IBM, NY, USA). One-sample Kolmogorov–Smirnov test was applied to the variables with quantitative values. The t-test was used for the variables of quantitative data that had a normal distribution and the Mann–Whitney test was utilized for the others. Proportions for categorical variables were compared using the Pearson chi-square test, although the Fisher’s exact test was used when the data were limited. Correlation analysis between the number admissions to urology clinics and the number of COVID-19 cases and deaths in the world and Turkey was performed. Multivariate linear regression analysis of predicting factors for urology outpatient clinic admissions and urological surgery was performed. The level of statistical significance was defined as p < 0.05.

## 3. Results

**Figure 1:**
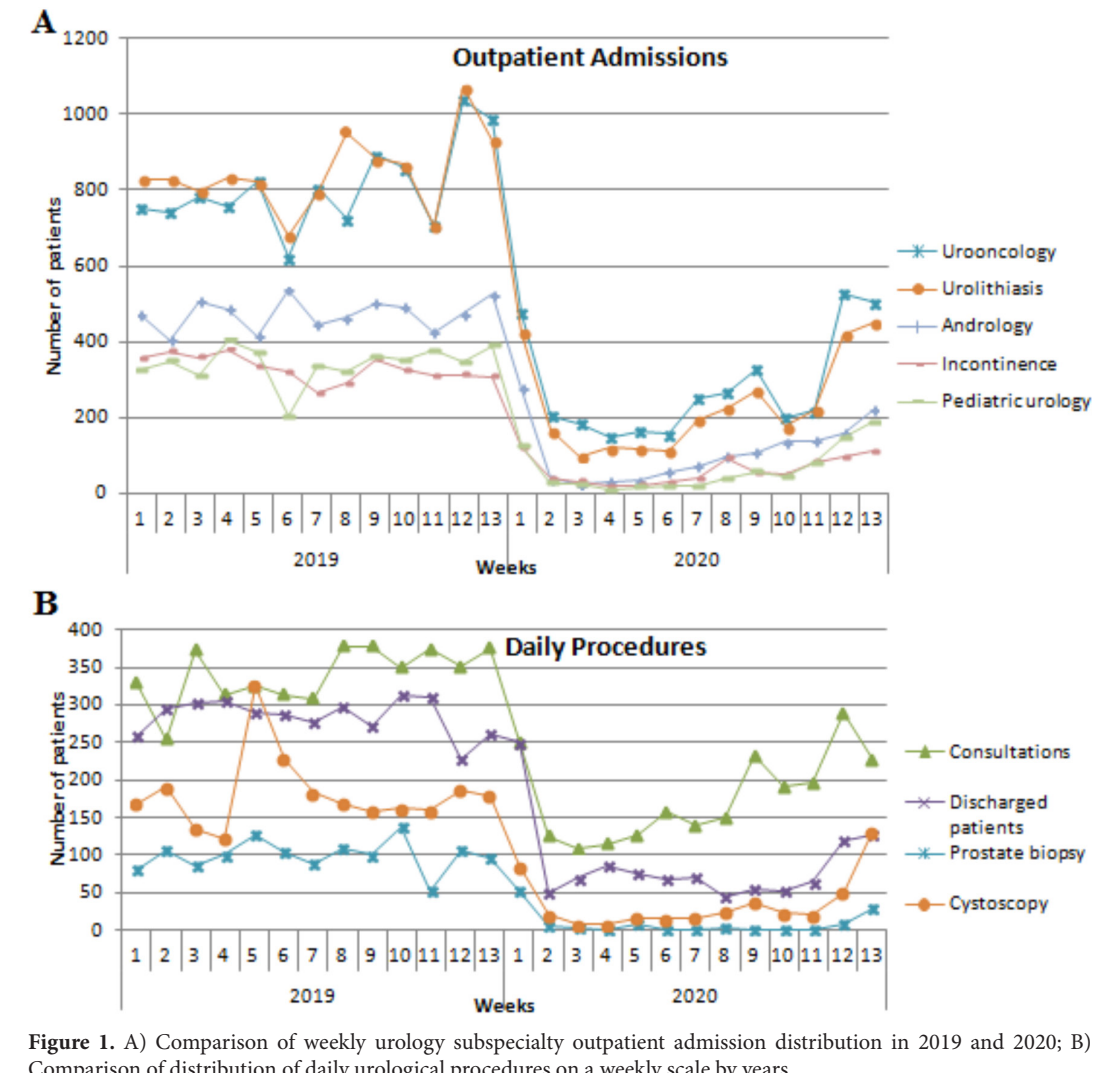


Outpatient clinic admission and surgical procedure distribution of patients are shown in Table 1. While 36,038 patients were admitted to the outpatient clinic in group 1, that number was 134,020 in group 2 (p < 0.001). There was a decrease in the number of patients admitted to all subspecialties in group 1; however, there was a significant proportional increase in urooncology (p < 0.001) and general urology (p = 0.011) (Figure 1A). Although group 1 had an increase in urolithiasis outpatient admission compared to group 2, this difference was not significant (p = 0.640). There was a significant difference in the number of consultations between the groups 1 and 2 (n = 2309 and 4425, respectively) (p < 0.001). Moreover, there was a dramatic decrease in the number of discharged patients (1132 vs 3706, in groups 1 and 2, respectively). A decrease of approximately 75% was observed in the total number of surgeries. While 3138 people had surgery in this quarter in 2019; this number dropped to 766 during the pandemic period (p < 0.001). Approximately 92% decrease was observed in prostate biopsy procedures (p < 0.001). Moreover, a decrease of approximately 80% was observed in the number of performed cystoscopies (p < 0.001) (Figure 1B). 

**Table 1:** Total numbers of outpatient clinic admissions, urological outpatient procedures, and surgical procedures during the 3-month period.

	Group 1 (March–June 2020)	Group 2(March–June 2019)	p-value
Total outpatient clinic medical care, n	36,038	134,020	<0.001
Subspecialty sections, n (%) Urooncology Urolithiasis Andrology Incontinence Pediatric urology Other (general urology)	3616 (10.1) 2984 (8.3) 1380 (3.8) 771 (2.1) 786 (2.2) 26,501 (73.5)	10,495 (7.8) 10,995 (8.2) 6127 (4.6) 4284 (3.2) 4454 (3.3) 97,665 (72.9)	<0.001 0.640 <0.001 <0.001 <0.001 0.011
Consultations, n (%) Emergency department Inpatient	2309 945 (40.9) 1364 (59.1)	4,425 2058 (46.5) 2367 (53.5)	<0.001 <0.001 <0.001
Discharged patients, n	1132	3706	<0.001
Surgical procedure, n	766	3138	<0.001
Prostate biopsy, n	109	1292	<0.001
Cystoscopy, n	455	2372	<0.001
Urooncological surgery, n (%)	238 (31.1)	716 (22.8)	<0.001
Endoscopic stone surgery, n (%)	163 (21.3)	755 (24.1)	0.104
Other surgeries	365 (47.7)	1667 (53.1)	0.007
TUR-B, n (%)	138 (18.0)	403 (12.8)	<0.001
TUR-P, n (%)	32 (4.2)	295 (9.4)	<0.001
Radical prostatectomy, n (%)	33 (4.3)	109 (3.5)	0.269
Radical cystectomy, n (%)	13 (1.7)	32 (1.0)	0.115
Radical inguinal orchiectomy, n (%)	25 (3.3)	51 (1.6)	0.003
Retroperitoneal lymph node dissection, n (%)	2 (0.3)	8 (0.3)	0.976
Radical nephrectomy, n (%)	20 (2.6)	61 (1.9)	0.246
Partial nephrectomy, n (%)	2 (0.3)	37 (1.2)	0.022
Radical nephroureterectomy, n (%)	4 (0.5)	11 (0.4)	0.491
Adrenalectomy, n (%)	1 (0.1)	4 (0.1)	0.983
Transvesical prostatectomy, n (%)	2 (0.3)	24 (0.8)	0.124
Fournier debridement, n (%)	8 (1.0)	12 (0.4)	0.021
Testicular tortion, n (%)	11 (1.4)	20 (0.6)	0.026
Internal urethrotomy, n (%)	28 (3.7)	141 (4.5)	0.307
Urogenital trauma, n (%)	5 (0.7)	8 (0.3)	0.002
Penile fracture, n (%)	7 (0.9)	8 (0.3)	0.008
Varicocelectomy/Hydrocelectomy, n (%)	9 (1.2)	179 (5.7)	<0.001
Transobturator tape, n (%)	0	45 (1.4)	-
Urethroplasty, n (%)	1 (0.1)	18 (0.6)	<0.001
Pyeloplasty, n (%)	1 (0.1)	13 (0.4)	<0.001
Ureteroneocystostomy, n (%)	0	9 (0.3)	-
Penile prosthesis, n (%)	0	2 (0.01)	-

TUR-B: Transurethral resection of bladder tumor; TUR-P: Transurethral resection of prostate

**Figure 2:**
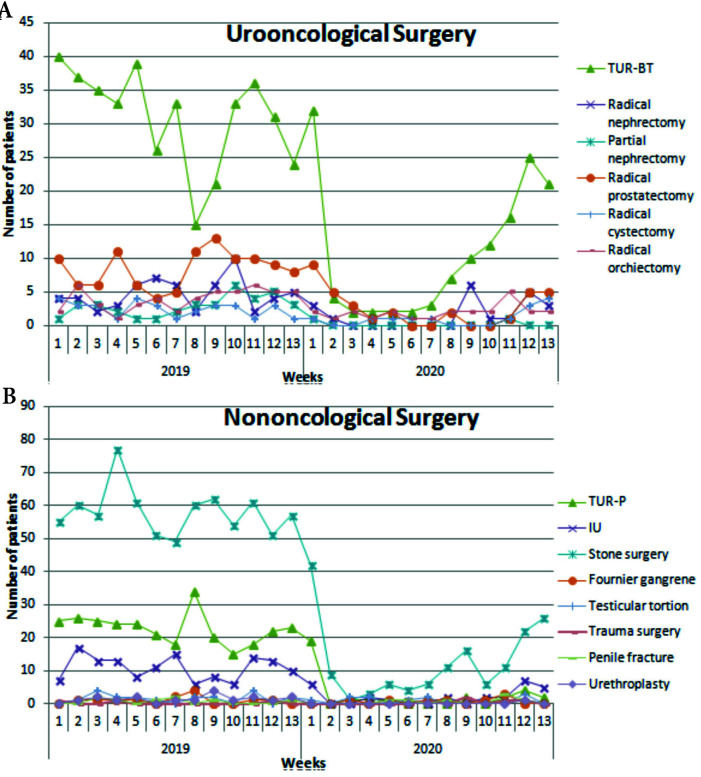


During the pandemic, the percentage of both the admissions to the urooncology outpatient clinic and the urooncological surgical procedures have increased. Endoscopic stone surgery rates were similar in both groups (p = 0.104) (Table 1). Urooncological surgical procedures, except for partial nephrectomy, have either increased proportionally or stayed stable (Figure 2A). In addition, we observed that the rate of urological emergency surgeries such as testicular torsion, urogenital trauma, and penile fracture increased statistically during the pandemic period (Figure 2B). The rates of reconstructive urological surgeries such as urethroplasty and pyeloplasty have decreased significantly (p < 0.001) (Table 1). 

**Figure 3:**
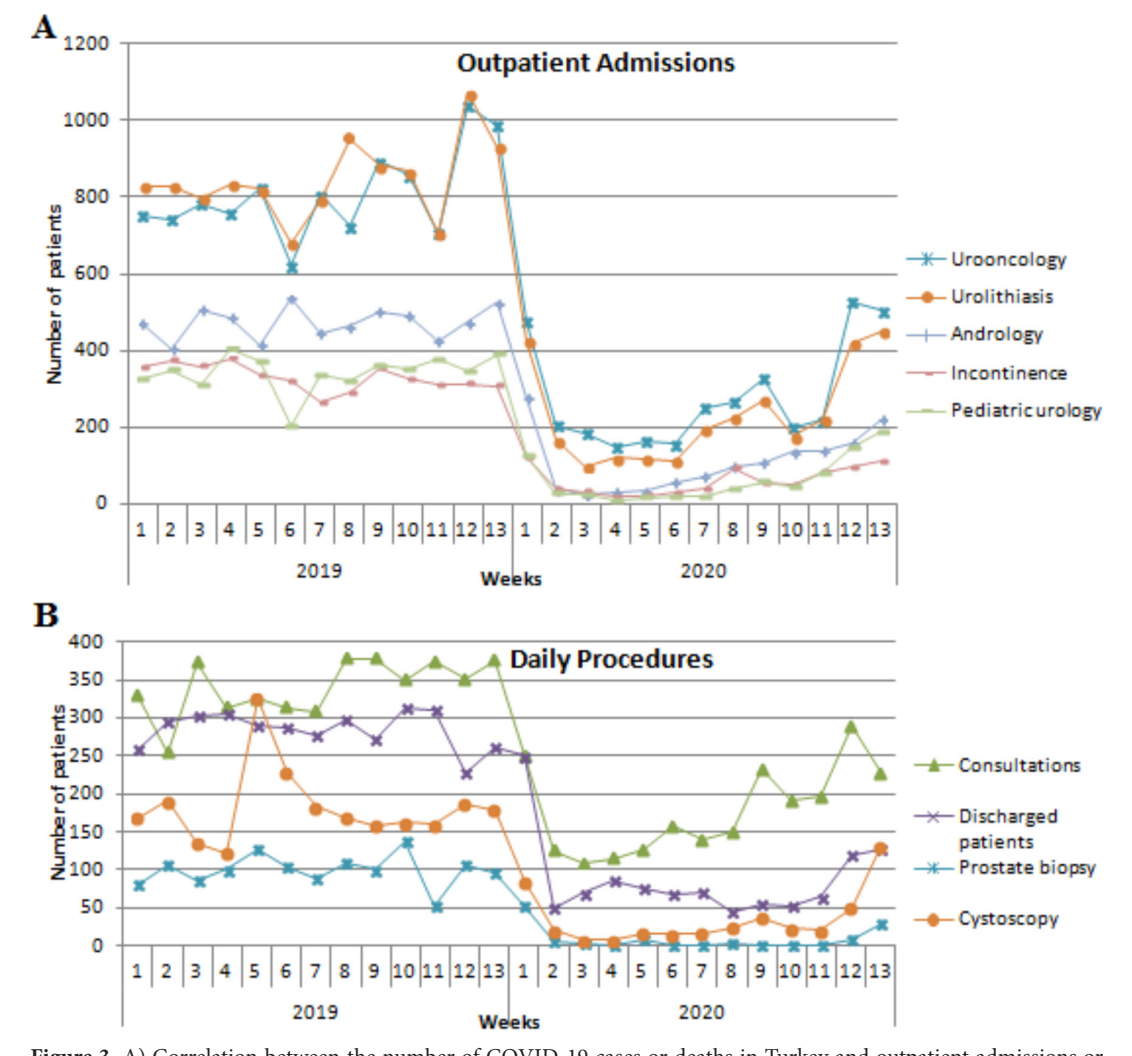


We detected a negative correlation between the number of admission to all outpatient clinics and COVID-19 cases or deaths in Turkey (p < 0.05). The same negative correlation was present for all surgical procedures and consultations (p < 0.05). In addition, the number of worldwide COVID-19-related deaths correlated negatively with urolithiasis, andrology, incontinence, general urology outpatient clinic visits, and all surgical procedures (Figures 3A and 3B). However, there was no correlation between the number of outpatient admissions and the number of worldwide cases (Table 2). 

**Table 2:** Correlation analysis between admissions and the number of COVID-19 case or deaths in Turkey and worldwide.

	Cases in Turkey	Deaths in Turkey	Cases in the world	Deaths in the world
Urooncology r p-value	–0.662 0.014	–0.633 0.020	0.21 20.488	–0.512 0.074
Urolithiasis r p-value	–0.708 0.007	–0.681 0.010	0.208 0.495	-0.569 0.042
Andrology r p-value	–0.737 0.004	–0.682 0.010	0.050 0.870	–0.665 0.013
Incontinence r p-value	–0.769 0.002	–0.718 0.006	0.130 0.673	–0.680 0.011
Pediatric urology r p-value	–0.660 0.014	–0677 0.011	0.363 0.223	–0.500 0.082
General urology r p-value	–0.715 0.006	–0.716 0.003	0.141 0.646	–0.624 0.023
Urooncological surgery r p-value	–0.751 0.003	–0.758 0.003	0.072 0.814	–0.689 0.009
Endoscopic stone surgery r p-value	–0.672 0.012	–0.654 0.015	–0.174 0.570	–0.708 0.007
Other urological surgery r p-value	–0.643 0.018	–0.653 0.015	–0.127 0.678	–0.686 0.010
Consultations r p-value	–0.669 0.012	–0.591 0.034	0.293 0.332	–0.478 0.099
Discharged patients r p-value	–0.384 0.195	–0.418 0.155	–0.371 0.212	–0.550 0.051

r: correlation coefficient

A multivariate linear regression analysis was performed to estimate the number of outpatient clinic admissions and operations using case and death numbers of Turkey and worldwide. As a result of the analysis, it was found that a meaningful regression model for outpatient clinic admissions, *F* (4, 8) = 2.86, p = 0.028, and 38% of the variance in the dependent variable (R2 adjusted = 0.38) were explained by the independent variables. Accordingly, the number of cases in Turkey, and number of deaths worldwide affect the number of outpatient clinic admissions in Turkey negatively and significantly. In addition, it was found that a meaningful regression model for urological surgery, *F* (4, 8) = 2.49, p = 0.020, and 33% of the variance in the dependent variable (R2 adjusted = 0.33) were explained by the independent variables. Accordingly, the number of cases in Turkey, and number of deaths worldwide affect the number of urological surgery in Turkey negatively and significantly (Table 3).

**Table 3:** Multivariate linear regression analysis of predicting factors for trend of urology outpatient clinic admissions and urological surgery.

Variables for outpatient clinic admissions	Multivariate model*						
	Unstandardized		Bootstrapping BCa 95% CI		Standardized		
	B	SEB	Lower bound	Upper bound	b	t	p-value
Cases in Turkey	–0.146	0.043	–0.240	–0.052	–0.718	–3.419	0.006
Deaths in Turkey	–5.034	1.497	–8.328	–1.739	–0.712	–3.363	0.006
Cases in the world	0.001	0.003	–0.005	0.008	0.155	0.520	0.614
Deaths in the world	–0.101	0.039	–0.187	–0.016	–0.617	–2.603	0.025
Variables forurological surgery	Multivariate Model**						
	Unstandardized		Bootstrapping BCa 95% CI		Standardized		
	B	SEB	Lower bound	Upper bound	b	t	p-value
Cases in Turkey	–0.005	0.001	–0.008	–0.001	–0.686	–3.131	0.010
Deaths in Turkey	–0.159	0.050	–0.270	–0.049	–0.691	–3.169	0.009
Cases in the world	–0.001	0.004	–0.011	0.008	–0.090	–0.298	0.771
Deaths in the world	–0.004	0.001	–0.006	–0.001	–0.704	–3.283	0.007

*The p-value of the model was 0.028.**The p-value of the model was 0.020.BCa 95% CI: The bias-corrected and accelerated bootstrap interval

## 4. Discussion

The COVID-19, caused by a novel SARS-CoV-2 virus, whose contamination dynamics are not known yet has begun to spread rapidly all over the world. The main symptoms of this disease are fever, dyspnea, cough, and gastrointestinal symptoms such as diarrhea, nausea, and vomiting [1]. In Turkey, the first COVID-19 case was detected on March 11, 2020, and since then the course of the disease was similar to other countries. After this date, it was reported that there was a significant decrease in the number of hospital admissions for urological or nonurological reasons all over the world. This decline was even observed in the number of emergency department admissions [2–5].

The differences in how daily urology practice is affected by the COVID-19 pandemic are likely to be influenced by regional variations in the severity of the COVID-19 outbreak. The pandemic resulted in changes in peoples’ general social behavior and also affected the day-to-day operations of the healthcare system [5–9]. The Turkish government enacted curfews for high-risk individuals, especially for people ≥65 years and restrictions on the general population on certain days. Even for patients over 65 years old, admission to the hospital was not restricted in any period. These protocols impacted individuals’ preferences, although they did not legally interfere with hospital admission. The functioning of the urology clinics located in the centers in İstanbul, which was one of the hard-hit areas, was also affected by these restrictions. In the early stages of the pandemic and afterwards, COVID-19 patients and non-COVID-19 patients were separated and treated in different parts of the hospital in the centers included in the study. Therefore, although the numbers of admissions decreased, daily urological procedures continued.

Most studies reported estimated 40%–80% decrease in outpatient services due to COVID-19 pandemic [6–8]. Clinicians tried restricting in-person visits during this period and provided health services to some of the patients via telemedicine [6,7]. Approximately 75% decrease was observed in overall number of patients visiting the participating outpatient clinics in İstanbul. We also observed a significant reduction in the number of admissions to 6 different outpatient centers during COVID-19 pandemic lockdown compared to the same period in 2019. This reduction was greater than half (>50%), especially for subspecialties such as urolithiasis (–72.9%), andrology (–77.5%), incontinence (–82.0%), pediatric urology (–82.4%), and urooncology (–65.5%). This reduction was statistically significant in all centers included in the study. Bozkurt et al. [9] reported a huge decrease of approximately 70%–80% in the number of outpatient, inpatient, total urological surgery, urooncological surgery, stone surgery, and emergent surgery in a multicenter study involving 51 centers from our country. These results show that COVID-19 greatly reduced the admission rate to urology clinics due to any reason. In addition to the decrease in the number of outpatient clinic admissions, the distribution of urology subsections showed that there was a change in the admission priorities. While a proportional increase was observed in urooncology outpatient admissions during the pandemic period, no change was observed in urolithiasis admissions. Moreover, while admission to other subspecialties of urology outpatient clinics decreased, application to general urology outpatient clinics tended to increase. A study from our country reported an increase in the number of ureteroscopic surgeries [5]. In another study, it was reported that urooncological and stone surgeries showed less decrease compared to total urological surgeries [9]. We also found a correlation between the decline in the number of admissions to urology outpatient clinics and increase in the number of COVID-19 cases and deaths in Turkey.

Numerous clinicians suggested postponing nonemergency urological procedures and the expert panel of European Urological Association provided recommendations for the management of surgical cases [10,11]. Gravas et al. [7] stated that more than 50% decrease was observed in all elective surgeries. Tyagi et al. [12] emphasized that during the pandemic, urological operations should be limited to high-risk urogenital cancers, urogenital traumas, urological emergencies, and patients with infected-obstructed urinary system. Although we thought that the lockdown would decrease the number of urological emergencies, there was no significant change in them compared to the same period last year; however, there was a proportional increase in urological emergencies during the pandemic. 

During the COVID-19 pandemic, the responsibility to decide which patient should be operated needs to be shaped by regional differences as well as guidelines and expert opinions. The results of the weekly review showed that the increase in the number of deaths in Turkey and worldwide also increased the avoidance of urological surgeries. This negative correlation was valid for all types of urological surgery in our study. This shows that patients avoided elective surgeries due to increasing COVID-19-related mortality.

The aim of postponing all nonemergency procedures was to ensure that COVID-19 patients had easy access to healthcare services as well as to reduce the risk of contamination to patients and healthcare providers [7,13,14]. Several studies have shown that telemedicine online visits were a safe and effective alternative to face-to-face outpatient clinic consultations for many urology patients [6–8,15]. Motterle et al. [16] reported that emergency urological consultations were also greatly affected by pandemic. In fact, Borchert et al. [8] reported that the proportion of those consultations that resulted in surgery decreased from 21.7% to 9.1% compared to the previous year. In our study, a statistically significant decrease was observed in the number and rate of patients consulted by the emergency department. However, it should be noted that delaying admission to the hospital for cases other than provocative urological pathologies such as severe colic pain, acute urinary retention, severe scrotal pain, penile fracture may cause various health problems in the future.

Measures taken to stop the spread of pandemic such as reducing the number of inpatients, postponing elective and low-risk cases can also affect urooncology patients. We concluded that in the pandemic period, when the number of surgeries decreased by approximately 75%, the proportion of urooncological surgeries has increased up to 31.1%. Although admissions to urooncology outpatient clinics have increased proportionally, decrease in the overall number of patients showed that even oncology patients avoided the hospital during the pandemic. In fact, the risk of morbidity and mortality due to COVID-19 of this group, which is one of the most frequent hospital visitors, was not low. Generally, they constitute the oldest group among the urological patient population [17]. Compared with the general population, the estimated incidence of COVID-19 in cancer patients was reported to be approximately 2.3 times higher [18]. In the pandemic period, fewer patient admissions, less diagnosis, and less medical treatment, and less surgical procedures were an expected result. Especially, prostate biopsy admissions were the most decreasing urological procedure. This clearly shows us the disruption in the diagnosis of prostate cancer, which is the most common urological cancer. The long-term oncological effects of this period will be revealed with the help of future studies. 

Patients may avoid hospitals due to various factors. In addition to the patients’ desire to protect their health, curfews imposed in the countries also impeded their hospital visits. However, hospital admission was inevitable for some patients. One of such subgroups was urinary tract stones, which could cause severe pain, complicated urinary infections, or hydronephrosis. An obstructed urinary collecting system should be decompressed by double-J stent or percutaneous nephrostomy [19]. In the current study, although the overall number of urinary system stone patients decreased, this decrease was not proportional. Similarly, the rate of endoscopic stone surgery did not decrease. The increase in the rate of admission in patients with complicated ureteral stones during the pandemic period in the study reported by Gul et al. [20] was due to the same reason that the rate of stone surgery in our study did not decrease.

To the best of our knowledge, there is no data about risk factors that cause avoidance from urology clinics for outpatient services, urological surgery, or other urological procedures. Our findings show that outpatient admissions, urological surgical procedures, and consultations were negatively correlated with the increase in the number of COVID-19 cases and deaths in Turkey. Moreover, there was a significant correlation between the decrease in all urological surgeries and increase in deaths in Turkey and worldwide. Multivariate regression analysis showed that outpatient admissions were affected by increased COVID-19 case and death numbers in our country, and deaths all over the world. Urological surgical procedures were affected by increased COVID-19 case numbers in our country and worldwide COVID-19-related deaths.

One of the limitations of the present study is that the clinical outcomes of this decreased rate of admission remain unknown and warrant long-term studies. Its retrospective design is another limitation. Moreover, the results may not be generalizable to other populations because they only contain data from İstanbul. However, considering Turkey in general, the inclusion of the highest-volume centers in the city, which was the epicenter of COVID-19 can be seen as the most important strength of our study. In addition, it is the longest interval study in the literature to date that examines the effect of COVID-19 pandemic on urological practice. Large sample size was also a strength of our study.

## 5. Conclusion

In our study, while the rate of urolithiasis remained stable during the pandemic, the rate of urooncology outpatient admissions and surgeries increased. A significant decrease was observed in other outpatient admissions, other urological surgeries, as well as daily urological procedures. Outpatient admissions and urological surgeries decreased significantly by increasing COVID-19 case numbers in Turkey and worldwide deaths. Urological surgical procedures were more affected by COVID-19-related deaths in Turkey and worldwide. This novel COVID-19 pandemic that the world is facing impacts even the urology practice. Perhaps, in the future, the effects of postponing certain urological procedures will be more evident. As urologists, we may be negatively affected by this situation with the patients in the postpandemic period.

## Financial disclosure

The authors declare that this study has received no financial support. 
